# Denaturing for Nanoarchitectonics: Local and Periodic UV-Laser Photodeactivation of Protein Biolayers to Create Functional Patterns for Biosensing

**DOI:** 10.1021/acsami.2c12808

**Published:** 2022-09-01

**Authors:** Augusto Juste-Dolz, Martina Delgado-Pinar, Miquel Avella-Oliver, Estrella Fernández, Jose Luís Cruz, Miguel V. Andrés, Ángel Maquieira

**Affiliations:** † Instituto Interuniversitario de Investigación de Reconocimiento Molecular y Desarrollo Tecnológico (IDM), Universitat Politècnica de València, Universitat de València, 46022 Valencia, Spain; ‡ Department of Applied Physics and Electromagnetism-ICMUV, Universitat de València, 46100 Burjassot, Spain; § Departamento de Química, Universitat Politècnica de València, 46022 Valencia, Spain

**Keywords:** biosensor, UV denaturation, immunoassay, non-specific binding, label-free, diffraction

## Abstract

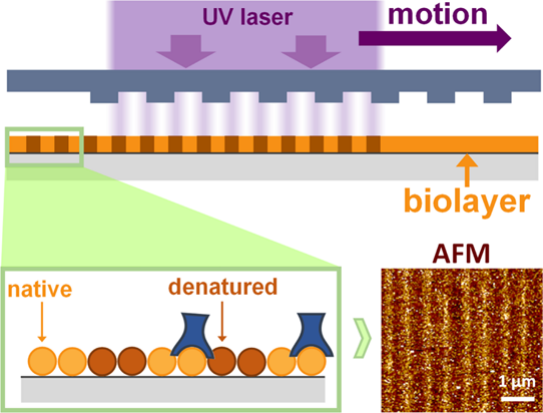

The nanostructuration of biolayers has become a paradigm for exploiting nanoscopic light-matter phenomena for biosensing, among other biomedical purposes. In this work, we present a photopatterning method to create periodic structures of biomacromolecules based on a local and periodic mild denaturation of protein biolayers mediated by UV-laser irradiation. These nanostructures are constituted by a periodic modulation of the protein activity, so they are free of topographic and compositional changes along the pattern. Herein, we introduce the approach, explore the patterning parameters, characterize the resulting structures, and assess their overall homogeneity. This UV-based patterning principle has proven to be an easy, cost-effective, and fast way to fabricate large areas of homogeneous one-dimensional protein patterns (2 min, 15 × 1.2 mm, relative standard deviation ≃ 16%). This work also investigates the implementation of these protein patterns as transducers for diffractive biosensing. Using a model immunoassay, these patterns have demonstrated negligible signal contributions from non-specific bindings and comparable experimental limits of detection in buffer media and in human serum (53 and 36 ng·mL^–1^ of unlabeled IgG, respectively).

## 1 Introduction

Nanoscience and nanotechnology are nowadays a fertile groundwork of materials and nanoscopic light-matter phenomena that provide unique solutions in endless scenarios. Within this field, the patterning of biomacromolecules points toward a promising scope in biomedical applications such as organ-on-a-chip,^1,2^ neuronal networks,^3−6^ drug delivery,^7^ and implant coatings^8^ among others. It also involves a particularly high impact in biosensing, where the biomolecular patterns are tailored to display nanoscopic phenomena to transduce biorecognition events.^9,10^ A crucial aspect in this scenario is the development of fast and large-scale methods to fabricate active nanostructures with a high geometrical accuracy.

A classical approach for structuring biomacromolecules is to place continuous biolayers onto prepatterned solid substrates,^11−13^ typically fabricated by photolithography,^14^ electron-beam lithography,^15^ dip-pen lithography,^16^ and laser interference.^17^ An alternative approach is to create nanostructures constituted by the biomacromolecules themselves on unstructured substrates. This strategy has been widely used to create microarrays for biosensing using techniques as contact and non-contact printing,^18^ photochemical surface chemistries,^19^ or patterned incubation masks.^20^ Among these nanostructuration techniques, microcontact printing (μCP) holds a noteworthy popularity for patterning biomolecules of different natures (proteins, nucleic acids, small molecules, etc.).^21^ μCP relies on the selective transfer of biomolecules from a nanostructured elastomeric stamp (typically made of polydimethylsiloxane) to a solid substrate just by contact. Even though μCP has demonstrated to be an excellent nanostructuration technique for biolayers in terms of versatility, simplicity, and cost-effectiveness, it presents some limitations, such as a moderate homogeneity of the resulting structures^22^ and a limited functionality of the patterned biomolecules.^23^

**Figure 1:**
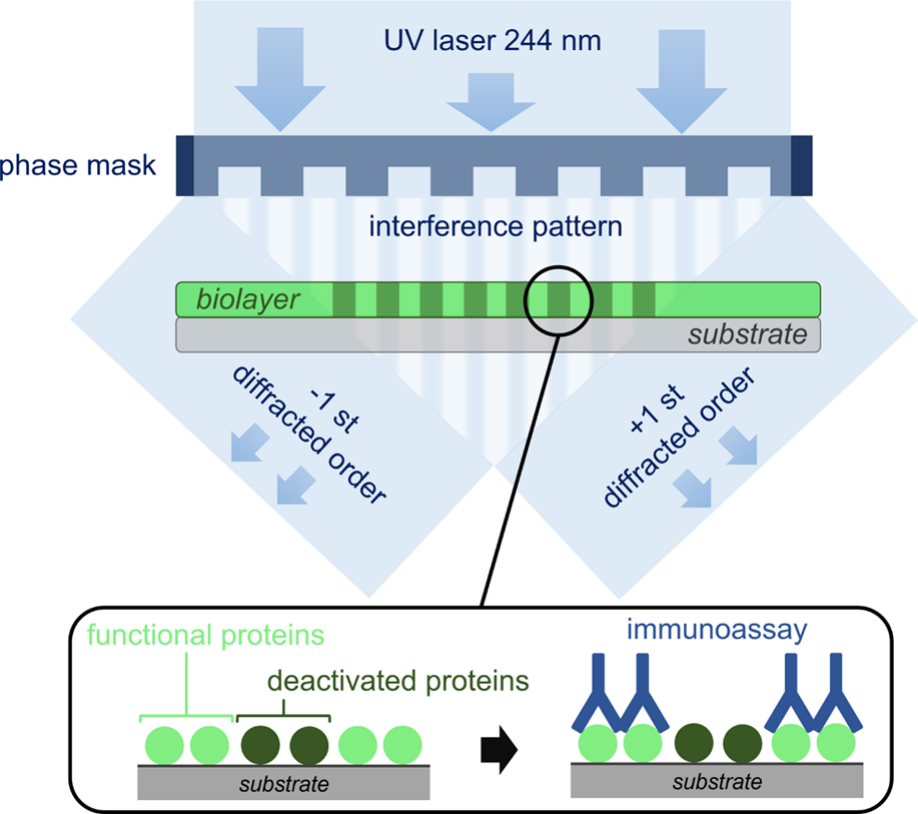
Scheme of the UV-induced selective protein deactivation process.

In this work, we present a method to create 1D periodic nanostructures of biomacromolecules on flat surfaces based on the local deactivation of protein biolayers assisted by UV laser. As schematized in Figure 1, the hypothesis behind this patterning strategy relies on irradiating surface-bound protein monolayers through a phase mask that generates an interferometric pattern of light on the biolayer. Proteins exposed to constructive interferences undergo a mild denaturation that impedes their functionality (without reaching ablation), and those exposed to the destructive interference keep their activity. Unlike standard UV photopatterning techniques typically based on photoresists, ablation, and inscribing refractive index variations on inorganic substrates,^26−28^ this approach aims to create patterns constituted by a periodic modulation of protein functionality and free of topographic contributions.

If these patterns of biomacromolecules are periodic at the nanoscale, they can interact with incident light beams and diffract them. Assessing this diffractive response provides useful information for the characterization of the structures. In addition, diffractive patterns of biomacromolecules have demonstrated to be a promising transduction system for biosensing.^29−34^ Among other features, they enable the development of miniaturized bioanalytical systems for real-time and label-free sensing, with a unique potential to minimize non-specific binding issues in the analysis of complex biological samples.^35^

Herein, we report the design and development of this patterning method for biomacromolecules based on periodic UV deactivation. First, the photofabrication parameters are explored and the structural features of the resulting protein patterns are characterized by microscopy and by assessing their diffractive response. Then, the homogeneity of the structures is investigated and compared with their counterparts fabricated by micro-contact printing. Finally, this work studies and reports the bioanalytical performance of these protein patterns for diffractive biosensing, investigates their potential to minimize non-specific binding contributions in biological samples, and provides insights into their multiplexing capabilities.

## 2 Results and Discussion

### 2.1 Photopatterning

The amount of light applied to the surface-bound bioreceptors is a key parameter in this photopatterning strategy since it will ultimately determine the rate of proteins that become deactivated and the magnitude of their denaturation.^25^ This aspect is herein investigated using a model immunoassay based on bovine serum albumin (BSA) protein probes and specific anti-BSA IgG targets.

After optimizing the surface concentration of the BSA protein biolayer (Figure S1), a range of UV fluences were experimentally assessed to explore their effect and to set up optimal conditions to create functional nanostructures. To modulate the fluences, both the emission power of the UV laser and the time of exposure on the protein surface were investigated. The time of exposure was controlled by the scan velocity of the UV laser along the phase mask, and the structural features of the resulting protein patterns were assessed by means of their diffractive response and their atomic force microscopy (AFM) profile.

Regarding the diffractive characterization, note that these patterns are periodic one-dimensional nanostructures conformed by alternated strips of active and inactive BSA proteins, where the active proteins will be able to bind their target IgGs, but the photodeactivated ones will not. As the relative amount of matter in the activated strips selectively increases because of the interaction with the target IgG, the periodic modulation becomes greater, and the diffraction efficiency increases too. As expected, neglectable diffraction efficiencies are experimentally observed in all the biolayers right after the photopatterning, regardless the irradiation fluence. Also, unstructured flat topographies are observed by AFM (Figure S2), suggesting that these fluences neither reach the threshold to create a periodic ablation of the biolayer or the glass surface nor lead to a severe protein denaturation that would introduce a significant periodic modulation of the refractive index. Instead, the results match the expected periodic mild denaturation of the surface proteins.

**Figure 2:**
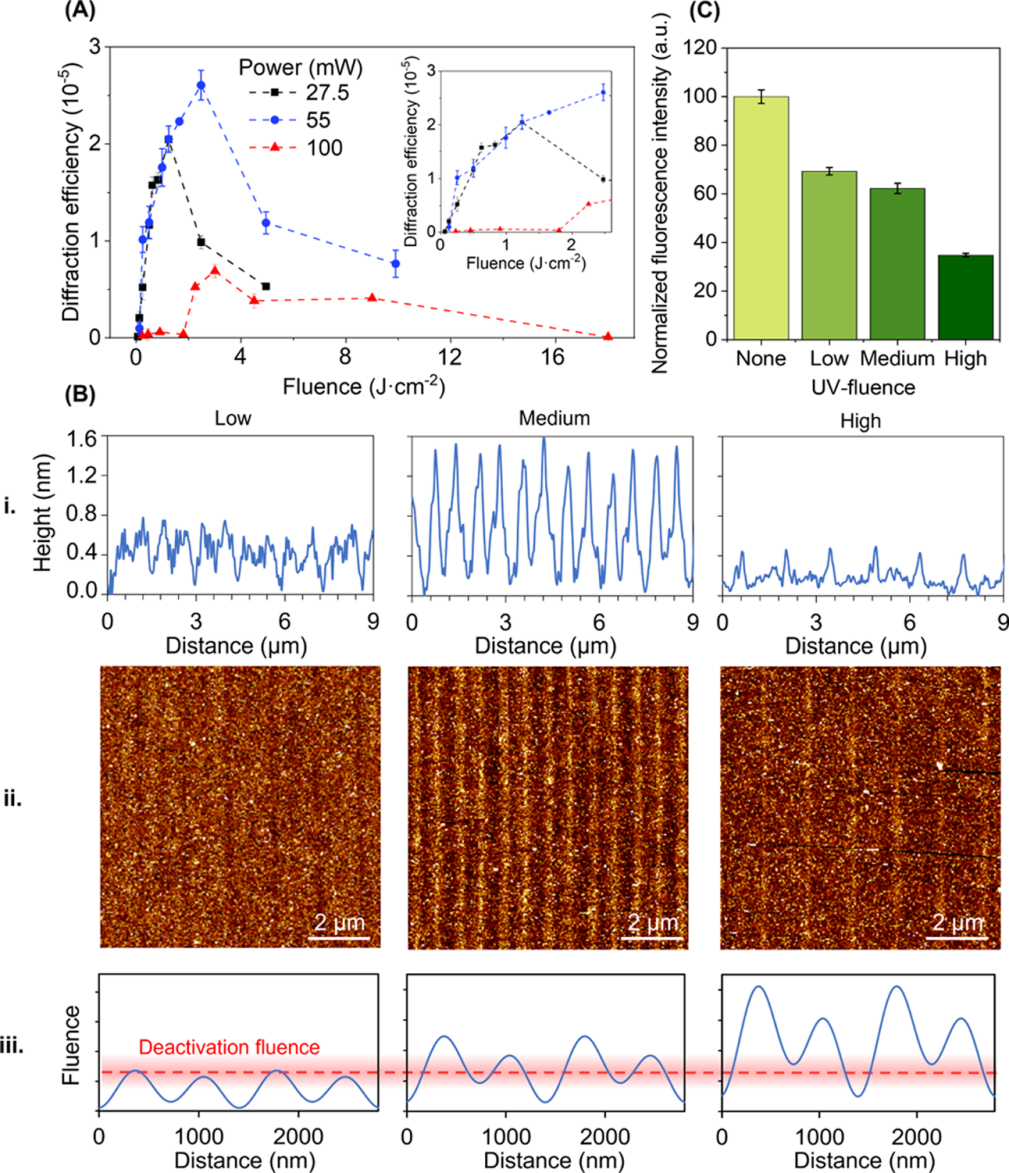
(A) Representation of the diffraction response of the BSA gratings obtained under different irradiation conditions. The inset shows a detail of the lower fluence range. (B) (i) Cross-section profiles of (ii) AFM images after incubating target anti-BSA IgG (10 μg·mL^–1^) onto protein layers irradiated with low (55 mW and 4.4 mm·s^–1^, 0.1 J·cm^–2^), medium (55 mW and 0.2 mm·s^–1^, 2.5 J·cm^–2^), and high (55 mW and 0.1 mm·s^–1^, 9.9 J·cm^–2^) fluences. Dark and bright colors indicate deep and high areas, respectively. See Table S1 for the corresponding topographic data. (iii) Scheme of the threshold deactivation fluence and the light profiles generated from the interference between the zeroth and first diffraction orders. (C) Fluorescence intensities from non-irradiated and UV-irradiated protein biolayers with low, medium, and high fluences after incubating fluorophore-labeled specific anti-BSA IgGs (10 μg·mL^–1^).

Then, to assess the deactivation profile, the irradiated biolayers were investigated after incubating a solution of specific target anti-BSA IgG (10 μg·mL^–1^) on them. Therefore, these IgGs should bind the proteins of the active strips but not the deactivated ones. A diffractive response is observed in all the cases (Figure 2A), which indicates the selective IgG binding according to the expected stripped pattern. The experimental results show different diffractive trends and topographic features for low, medium, and high irradiation fluences as discussed below.

As shown in Figure 2A, the low-fluence range (from 0 to about 1.5 J·cm^–2^) displays a low diffractive response that increases together with the fluence. It indicates that the aimed periodic protein deactivation also takes place at these fluences, although it involves a lower height modulation. In fact, irradiation fluences as low as 62 mJ·cm^–2^ are enough to create a pattern. On the other hand, the diffractive responses of the patterns created by different laser powers (27.5 and 55 mW) overlap in this low-fluence range, whereas this is not the case for the rest of the curve. This observation suggests that the biolayer presents nonlinear response to the laser power and the scan velocity, and therefore, both parameters must by optimized simultaneously.

An optimal range is shown at a medium fluence of 1.5–4 J·cm^–2^ (Figure 2A). In particular, the maximal diffractive response is obtained in protein patterns created at 2.5 J·cm^–2^ with a laser power of 55 mW, and a dropping trend in the diffraction efficiency is observed beyond this medium range in all the cases.

These results indicate that the highest rate of denaturation between active and deactivated strips corresponds to medium irradiation fluences, and this observation is supported by the topographic characterization. The biolayers exposed to medium fluences display greater height modulations after the immunoassay than those created at low and high fluences (Table S1). Also, as shown in Figure 2B(i,ii), the target anti-BSA IgGs selectively bind to active protein strips, generating a homogeneous, periodic, and grooved structure.

Regarding the period of the biolayer patterns, the one expected for the employed phase mask (710 nm) is obtained in all the cases, as measured by AFM (Figure 2A and Table S1). A contribution of a double-length period (around 1420 nm) is also observed in the diffractive response and in the AFM scans and comes from the two effects schematized in Figure 2B(iii). One of them is a deactivation fluence of a relatively wide range, rather than a narrow value. The other one is a non-negligible contribution of the zeroth diffraction order of the phase mask, which interferes with the first orders and generates a sinusoidal light profile on the biolayer constituted by alternated lobes of higher and lower intensity. Although only a power contribution of about 3% is expected from the zeroth order,^36^ the experimental results show that it can involve a significant impact in the resulting protein pattern. The interaction of these two effects can also explain the deviation in the duty cycle measured by AFM (Table S1), around 60 and 40% for low and high fluences, respectively. This issue can be minimized by selecting proper irradiation parameters (laser power and scan velocity), and our experimental results show that a minimal presence of this double period and an optimal duty cycle of around 50% are simultaneously obtained in the structures fabricated at medium fluence.

Regarding the changes undergone by the surface-bound proteins due to the irradiation, proteins absorb UV light thanks to the side chain of the aromatic amino acids. This excitation can generate an electron flux that induces the breakage of disulfide bridges and irreversibly modify the three-dimensional conformation of the protein.^24,25^ On one hand, the formation of disulfide bridges requires two nearby cysteines for their side chains to interact. On the other, among the aromatic amino acids, tryptophan has the highest absorption coefficient in the near UV region and plays a central role in the electron transfer for the photolytic cleavage of nearby disulfide bridges.^24,37^ In the case of the BSA proteins used in this study, they are constituted by 607 amino acids, with 3 tryptophans and 34 cysteines forming disulfide bridges (Figure S3), which are the main compounds responsible for the photopatterning process herein studied.^37,38^

This UV-induced disulfide bridge disruption may modify the three-dimensional conformation of the protein. However, these periodic conformational changes are not experimentally detected in the AFM topographic characterization (Figure S2), presumably given their negligible contribution in the resulting height modulation of the pattern. On the other hand, it must be highlighted that after the irradiation at medium fluence, the patterned protein biolayers do display a minute diffractive signal. Although this diffraction efficiency is about 3 orders of magnitude lower than the corresponding one after binding target antibodies (1.1 × 10^–8^ before and 2.8 × 10^–5^ after the incubation of 10 μg·mL^–1^ of specific IgGs), these results suggest that irradiated proteins undergo a conformational change that slightly modifies their refractive index.

To assess the protein deactivation rate, we also measured the fluorescence intensity after incubating specific anti-BSA IgGs labeled with a fluorophore. Instead of structural information of the patterns, these measurements provide information about the overall deactivation rate of the biolayer, where a higher fluorescence intensity indicates a greater amount of bound targets and therefore a lower deactivation. As shown in Figure 2C, when a higher fluence is applied, greater overall deactivation is obtained and therefore, a lower fluorescence signal is acquired. This observation complements the abovementioned characterization and supports the hypothesis of this structuration strategy.

From these results, protein patterns fabricated by a fluence of 2.5 J·cm^–2^ (55 mW laser power and 0.2 mm·s^–1^ scan velocity) were selected to further investigate this patterning method. It is worth highlighting that for these patterning conditions, about 20 mm^2^ of optically active structures can be patterned in less than 2 min. Furthermore, once fabricated and stored at 4 °C, these protein patterns have shown to keep their optical and binding functionality for more than 30 days (Figure S4).

### 2.2 Structural Homogeneity

Once fabricated, the overall homogeneity of the obtained protein patterns was assessed by means of their diffractive response. Herein, these results are experimentally compared with those obtained by micro-contact printing (μCP) because this is an important technique widely employed to pattern biomacromolecules and also used to create diffractive protein structures.^9,23,29,32^

**Figure 3:**
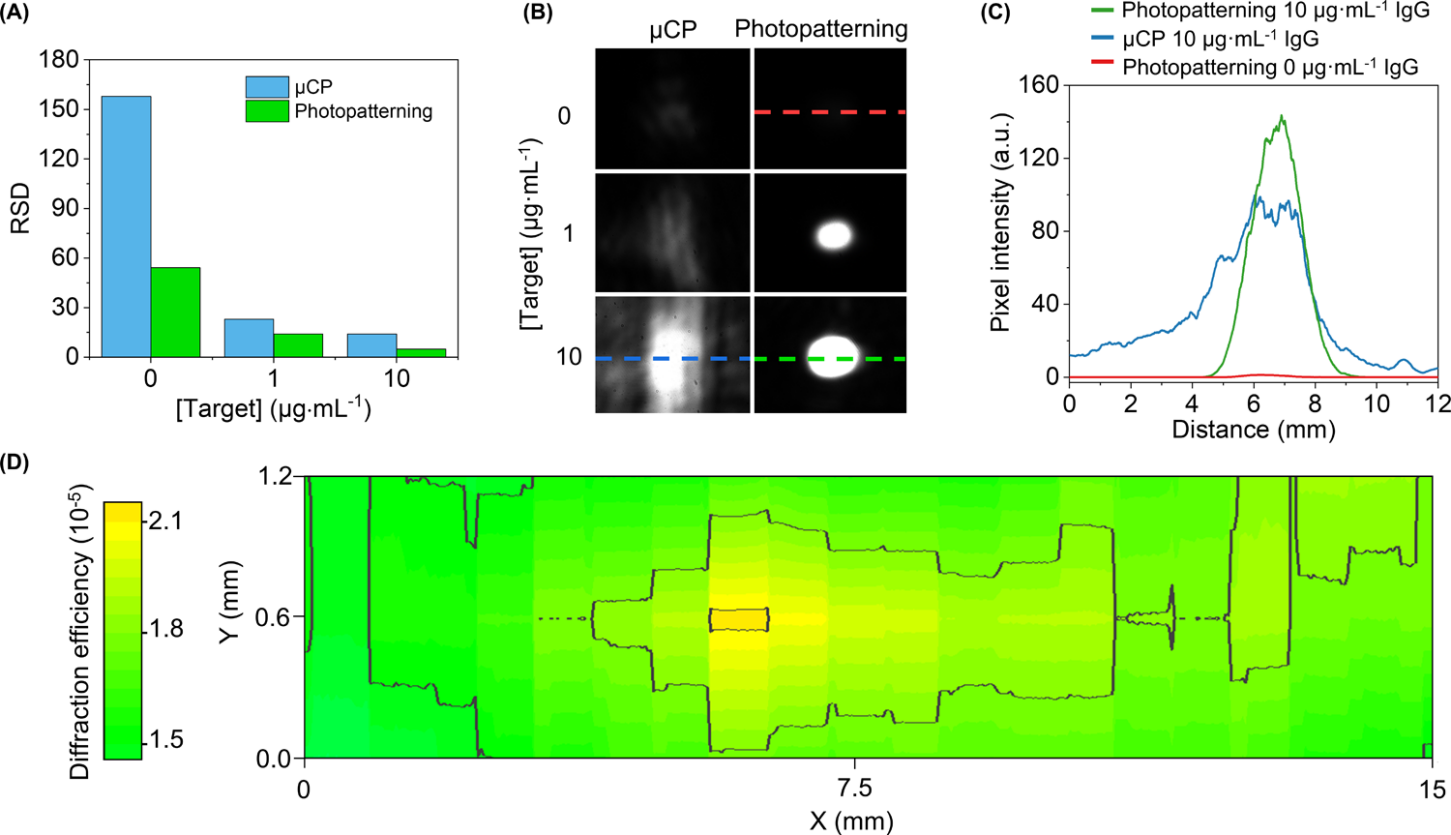
(A) Homogeneity assessment. RSD values of the diffraction efficiency (three replicates) and (B) images of the first-order diffracted spots obtained with BSA patterns fabricated by photodeactivation and μCP after the incubation of different concentrations of specific anti-BSA IgG in buffer solution. (C) Cross-section profiles of the first-order diffracted spots, where the profile direction along the spot is indicated by the dashed line in Figure 3B. See Figure S5 for a zoomed view of the cross-section for the photopatterned biolayer after the incubation of 0 μg mL^–1^ of anti-BSA. (D) Diffraction efficiency mapping of the first diffracted order of a photopatterned biolayer incubated with 10 μg mL^–1^ of specific anti-BSA IgGs and the corresponding cross-section indicated as a dashed line.

First, the repeatability of the gratings was assessed by means of the relative standard deviation (RSD) of the diffraction efficiency obtained after the incubation of specific anti-BSA IgG targets. As shown in Figure 3A, the RSD value for the photopatterned biolayers is about 2-fold better than the one displayed by μCP. This improvement is especially significant in blank samples (0 μg·mL^–1^ of IgG) since the diffracted signals of the photopatterned BSA gratings are negligible (Figure 3B,C). Therefore, this effect impacts on the experimental noise rates and will ultimately affect the detection and quantification limits for biosensing.

Then, the overall homogeneity of the patterned biolayers was also assessed by means of the shape of the diffracted light spots. Structural irregularities and deformations scatter the incident light and even lead to period changes that distribute the diffracted beam on a wider and more irregular area.^39^ As shown in Figure 3B,C, the diffracted spots from biomolecular gratings obtained by μCP are typically defined by an uneven and wider distribution. On the other hand, the diffracted spots generated by the biolayers patterned by this photodeactivation strategy are constituted by a well-defined Gaussian-like profile that concentrates the diffracted light in a regular area, which provides insights into the great homogeneity of these structures.

The homogeneity of the resulting biomolecular structures was assessed by mapping their diffractive response along the patterned area (Figure S6). As shown in Figure 3D, large areas of optically active protein nanostructures can be patterned with this method. The horizontal (*x*) dimension in this plot corresponds to the motion direction of the laser during the patterning, and the other (*y*) one corresponds to the vertical expansion of the laser beam by a cylindrical lens included in the patterning setup (Figure S7). In this first approach, an RSD of 16% is obtained from the diffractive mapping of the patterned strip of 15 × 1.2 mm, which will be selected as the sensing area in the next steps of this study.

### 2.3 Immunosensing

The abovementioned disulfide bridge cleavages undergone by the surface-bound biolayers exposed to constructive UV interferences can modify the protein parts that act as epitopes in antibody-mediated biorecognition events, and these changes can affect the subsequent binding processes of specific antibodies. To explore the biosensing capabilities of this approach, we used a representative immunoassay based on BSA probes and specific anti-BSA IgGs as targets. A whole antiserum is used as anti-BSA in this study, which provides more insights into the applicability of these photopatterned biolayers. This antiserum contains specific antibodies that are polyclonal, thus involving a wide range of paratopes for different lineal and conformational epitopes.

To assess the effect of the UV irradiation on the binding process, BSA patterns were created, and their response was experimentally measured after the incubation of a single concentration of anti-BSA (10 μg·mL^–1^). Using labeled secondary antibodies, it is observed that strong irradiations substantially hinder the subsequent binding of specific antibodies (Figure S8), and this effect increases together with the fluence applied in the photopatterning (Figure 2C). Furthermore, when comparing the topography before (Figure S2) and after (Figure 2B, medium fluence) the antibody incubation, a selective height growth following the photopatterned striped structure is observed. This local and periodic antibody binding is also confirmed by the dramatic increase of the diffraction efficiency observed after the incubation (Figures 3C and S5). All these results confirm that the UV-induced modifications undergone by the surface-bound proteins hamper the activity as epitopes for the subsequent biorecognition events with antibodies and that this binding follows the periodic structure created in the photopatterning.

**Figure 4:**
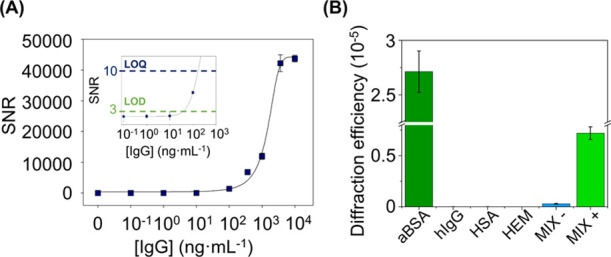
(A) Immunoassay calibration curve. Experimental data fitted to a sigmoidal regression (four-parameter logistic). The inset zooms in on the detection and quantification limits. (B) Diffraction efficiencies achieved after incubating 10 μg·mL^–1^ of specific IgG targets (aBSA); human IgGs (hIgG); human serum albumin (HSA); hemoglobin (HEM); and a mixture of hIgG, has, and HEM without (MIX−) and with (MIX+) 10 μg·mL^–1^ of anti-BSA in PBS-T buffer.

To further characterize the capabilities of these photopatterned biolayers as diffractive transducers for biosensing, their diffractive response upon the incubation of a range of antibody concentrations was investigated. As shown in Figure 4A, the system displays a well-correlated calibration curve (*R*^2^ = 0.999) that fits the expected trend for this biorecognition event. From these results, experimental detection and quantification limits of 53 ng mL^–1^ (0.4 nM) and 164 ng mL^–1^ (1.1 nM) of anti-BSA IgG are inferred, respectively. These are promising values for this novel patterning approach, determined under experimental and label-free conditions, which are in the range of other recent label-free optical approaches in the state of the art (Table S2).

An important issue in label-free biosensing is the signal contribution of non-specific bindings (NSBs), an undesired phenomenon that takes place specially in the analysis of biological or other complex samples,^35,40^ which contain many molecules at different concentrations that are prone to adsorb non-specifically on the sensing surface and generate signals that cannot be discriminated from the probe-target biorecognition events. A particular feature of diffractive biosensing approaches is their potential to avoid signal contributions from NSB. It relies on the fact that only the binding events that meet the periodicity of the patterned biolayer create a periodic modulation that modifies the diffraction efficiency of the nanostructure as it happens for the recognition between the patterned active probes and their targets. However, the adsorption of non-specific binders on the biolayer follows a random and not periodic distribution and therefore does not modify the diffraction efficiency.^30^

A positive aspect to favor the randomness of the NSB process is to keep the same chemical composition on both kinds of strips of the patterned biolayer. Therefore, the non-specific binders present the same tendency for both parts of the pattern and they become evenly distributed as desired to avoid NSB signal contributions. This is the case for the structures herein investigated, where activated and deactivated strips are constituted by the same biomacromolecule, only differentiated by a mild modification that changes its binding capability.

As a first step to explore the ability of this approach to minimize NSB signal contributions, the diffractive response upon the incubation of high concentrations (10 μg·mL^–1^ in buffer solution) of non-specific binders typically found in serum was assessed. As observed in Figure 4B, negligible signals compared to the one for the binding of specific anti-BSA IgG at the same concentration are obtained. In addition to the NSB issue, note that this experiment also points out the analytical selectivity of the assay.

**Figure 5:**
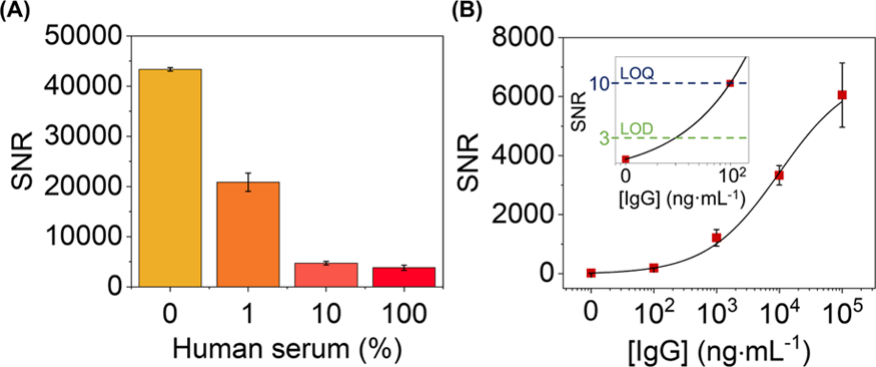
(A) SNR values achieved after incubating different dilutions of human serum (in PBS-T) spiked with specific IgG (10 μg·mL^–1^). (B) Immunoassay calibration curve performed in pure human serum. Experimental data fitted to a sigmoidal regression (four-parameter logistic).

Then, we explored the response of the system under a range of dilutions of a commercial human serum containing 6.5 × 10^4^ μg·mL^–1^ of non-specific proteins, 1025 μg·mL^–1^ of triglycerides, and 1600 μg·mL^–1^ of cholesterol, which are potential non-specific binders. On one hand, all these serum incubations displayed negligible changes in the diffractive response of the biomolecular pattern, which points out that unwanted additive signal contributions from NSB are avoided. On the other hand, the diffraction efficiency decays with the concentration of non-specific binders when target anti-BSA IgGs are spiked in these serum dilutions, as shown in Figure 5A. Note that the concentration of non-specific binders in this real sample is many orders of magnitude larger than the one of specific targets. It may lead to steric clashes and hindered diffusive processes that decrease the availability of free patterned probes to interact with the specific targets. Interestingly, the results show that together with this signal decrease, the experimental noise value undergoes a dramatic decay too, and as a result, favorable signal-to-noise ratios (SNRs) are also obtained under these high NSB conditions. As shown in Figure 5B, great SNRs and a well-correlated calibration curve (*R*^2^ = 0.998) are obtained in pure human serum. From these results, the experimental detection and quantification limits in pure serum (36 and 100 ng·mL^–1^, respectively) reached similar values to those obtained in buffer.

**Figure 6:**
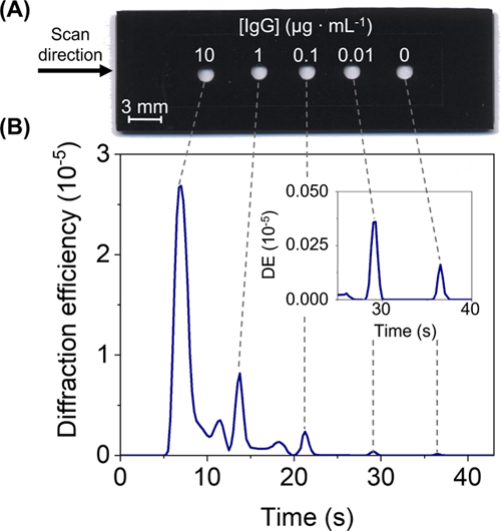
Multiplexed scanning. (A) Top-view photograph of a glass slide with a patterned protein biolayer after attaching the incubation mask. (B) Cross-section of the signal profile acquired with the diffractive scanning after incubating the IgG concentrations indicated above on each spot in the buffer.

As an exemplary approach to provide preliminary insights into the implementation of these photopatterned biolayers in detection schemes for multiplexed biosensing, the mapping setup commented above (Figure S6) was employed to automatically scan the diffractive response of different assays in a single measurement. For this, incubation masks of adhesive film were attached on the slides after the photopatterning and used to create several sensing areas where different target concentrations were incubated. As shown in Figure 6, an array of multiple sensing spots can be easily created, and their response is measured in less than 40 s. Beyond this first approximation, arrays containing a larger number of sensing spots can be easily arranged to automatically quantify many targets in a single assay with these photopatterned biolayers.

## 3 Conclusions

This work introduces a patterning method for biolayers based on the local deactivation of surface-bound proteins by UV-laser irradiation. The results support the design, optimization, characterization, and fabrication of one-dimensional periodic distributions of biomacromolecules with label-free biosensing capabilities. The proteins that are exposed to the UV-radiation conditions become deactivated but not removed from the substrate, thus producing protein patterns free of topographic contributions but constituted by a periodic deactivation of the protein activity. This method enables a fast fabrication of large areas of homogeneous protein patterns, whose analytical capabilities as diffractive optical transducers for biosensing are demonstrated by calibration curves with a representative immunoassay in label-free format. The resulting photopatterned protein nanostructures present a particular potential to avoid non-specific binding issues in the direct analysis of complex biological environments. In addition to providing insights into multiplexed biosensing, these results also introduce the basis for the prospective implementation of this photodenaturation-based patterning principle in alternative laser technologies and applications.

## 4 Experimental Section

### 4.1 Materials

Sodium phosphate buffer (PBS, 8 mM Na_2_HPO_4_, 2 mM, 137 mM NaCl, 2.7 mM KCl, pH 7.4), PBS-T (PBS with polysorbate 20 0.05% v/v), and carbonate–bicarbonate buffer (15 mM Na_2_CO_3_, 34 mM NaHCO_3_, pH 9.6) were prepared with purified water (Milli-Q, Millipore Iberica, Darmstadt, Germany) and filtered through 0.2 μm polyethersulfone membranes (Merck, Darmstadt, Germany). BSA, polysorbate 20 (Tween 20), anti-BSA IgG produced in rabbit (whole antiserum), human serum albumin (HSA), human IgG, hemoglobin, goat anti-rabbit antibodies labeled with 5 nm gold nanoparticles, and silver enhancers were supplied by Sigma-Aldrich (Madrid, Spain). An Alexa Fluor 647 conjugation kit was from Abcam (Cambridge, United Kingdom). Polydimethylsiloxane (PDMS) Sylgard 184 was supplied by Dow Corning (Wiesbaden, Germany). Human serum obtained by centrifugation of a pool of blood samples (type AB) from male donors was provided by Sigma-Aldrich (Madrid, Spain). Glass slides (25 × 75 × 1 mm) were purchased from Labbox (Barcelona, Spain).

Glass slides were washed three times by sonication in ethanol (30% in water, 5 min) and dried under a stream of air. Then, protein solutions in carbonate buffer (500 μL, 25 μg·mL^–1^) were incubated overnight on the glass slides at 4 °C (Figure S1). Finally, glass slides were rinsed with deionized water and dried by air stream.

### 4.2 Patterning

The periodic deactivation of the protein layers was performed by an optical setup described in Figure S7. Basically, it consists of a continuous wave UV laser (Fred doubled argon laser, 244 nm, 100 mW adjustable power) (Coherent, Santa Clara, California, USA) that, after passing through a phase mask (±1 order working principle, 1420 nm period, 2.5 cm length, duty cycle 50%) (Ibsen Photonics, Farum, Denmark), irradiates a protein biolayer created on glass slides. The interference of the +1st and −1st order creates a light intensity pattern which interacts with the biolayer. A cylindrical lens (divergent lens, 2 cm focal length) (OptoSigma, Santa Ana, California, USA) is included between the laser and the phase mask to expand the beam along the vertical direction. The power of the laser is measured with an optical power meter Mentor M10 (Scientech-Inc, Boulder, Colorado, USA). In this setup, the glass slides with the biolayer together with the phase mask are placed onto an automatic positioning system (Physik Instrumente GmbH, Karlsruhe, Germany) that moves the incident beam over the samples to be irradiated along the horizontal direction at a controllable velocity.

The irradiation fluence is calculated as (*P*·*W*)/(*A*·*V*), where P is the power of the laser (27.5, 55, and 100 mW), *W* is the width of the laser spot on the biolayer along the translational direction of the positioning system (0.1 cm), *A* is the area of the laser spot on the biolayer (0.1 cm^2^), and *V* is the velocity of the positioning system (from 6 × 10^–3^ to 0.4 cm·s^–1^).

### 4.3 Characterization

The diffractive measurements were performed in a transmission configuration using a simple optomechanical setup illustrated in Figure S7. The glass slides with protein nanopatterns were set to be orthogonally irradiated by a collimated and attenuated (50%) 532 nm laser source (100 mW, MGL-III-532/1, CNI, Changchun, China). The intensity of the diffracted beams was registered using a monochromatic CMOS camera (Edmund eo-1312m, York, UK) and photosensors created from planar silicon photodiodes (SLC-61N2, Silonex Inc., Montreal, Canada). The diffraction efficiency of the protein patterns, that is, analytical signal, was calculated as the quotient between the intensity of the first and zeroth diffraction orders. RSD values for each sample were calculated as the ratio between the standard deviation and mean values of three diffraction measurements performed within the patterned area.

These results were compared to protein nanopatterns fabricated by microcontact printing as described elsewhere.^34^ Basically, BSA solutions (250 μg·mL^–1^ in PBS) were incubated onto the nanostructured surface of the PDMS stamps for 160 min, and after washing them with deionized water and drying them under air stream, they were stamped onto glass slides for 20 min. Finally, the glass slides were washed and dried as before.

The mapping of the diffraction efficiency along the whole area was performed with a custom scanning system that sequentially moves the surface and collects the optical signals, as described elsewhere.^41^ Two photosensors were incorporated in this case to measure the transmitted zeroth and first orders (Figure S6), and RSD values were calculated from the diffraction efficiency of all the pixels within the sensing area (20 × 1.2 mm). The resulting data from the scans were smoothed with a Savitzky–Golay filter (second-order polynomial, 30 points).

For the fluorescence measurements, IgG targets were labeled with an Alexa Fluor 647 and incubated on the patterned biolayers. Then, fluorescence images were acquired with a custom fluorescence CCD camera (Retiga EXi camera, QImaging Inc., Burnaby, Canada) and an oblique LED source (Toshiba TLOH 157 P Toshiba, Tokyo, Japan). The resulting data were analyzed with the GenePix Pro 6.0 software (Molecular Devices, San José, California, USA).

The topography of the nanostructures was analyzed by atomic force microscopy (AFM) using a Bruker Multimode 8 microscope (Bruker, Massachusetts, USA) and with RFESPA probes (MPP-21120-10 Bruker) before and after incubating specific targets. AFM images were analyzed using Nanoscope software. To calculate the averaged cross-section profiles, all images were flattened using a first-order polynomial fitting and the height of every data row along the longitudinal direction of the pattern strips was averaged. From these cross-sections, the height modulation is calculated as the average height of the deactivated strips subtracted to the one of the active strips. The duty cycle is calculated as the percentage of the averaged width of the active strips with respect to the period.

### 4.4 Biorecognition Assays

To perform the immunoassays, 500 μL of target IgG (anti-BSA) solutions in PBS-T and human serum were incubated onto the photopatterned protein (BSA) biolayers for 15 min at room temperature. Then, each slide was rinsed with PBS-T and deionized water and dried under air stream. The same procedure was followed for the fluorescence assays, but in this case, the target IgGs were labeled with a fluorophore (Alexa Fluor 647) before the assay.

Three replicates of each condition were measured to calculate averaged and standard deviation values. Noise was appraised as the standard deviation from 10 blank measurements (0 μg·mL^–1^ of target IgG incubated on 10 different nanostructures) and employed to determine SNRs. The limits of detection and quantification were calculated as the concentrations associated to SNR = 3 and SNR = 10, respectively, from the linear interpolation in the experimental calibration curves.

## Supporting Information Available

Optimization of the BSA coating, AFM results right after the patterning, compositional and structural data of BSA, results on the time stability of the protein patterns, zoomed view of Figure 3C, schemes of the setups for diffractive measurements and for photopatterning, effect of strong irradiations, summary of topographic features obtained by AFM, and comparison with recent approaches in the state of the art (PDF)

## References

[ref1] Arrabito G.; Ferrara V.; Bonasera A.; Pignataro B. Artificial Biosystems by Printing Biology. Small 2020, 16, 1907691 10.1002/smll.201907691. 32511894

[ref2] Park J. Y.; Jang J.; Kang H. W. 3D Bioprinting and Its Application to Organ-on-a-Chip. Microelectron. Eng. 2018, 200, 1–11. 10.1016/j.mee.2018.08.004.

[ref3] Lantoine J.; Procès A.; Villers A.; Halliez S.; Buée L.; Ris L.; Gabriele S. Inflammatory Molecules Released by Mechanically Injured Astrocytes Trigger Presynaptic Loss in Cortical Neuronal Networks. ACS Chem. Neurosci. 2021, 12, 3885–3897. 10.1021/acschemneuro.1c00488. 34614352

[ref4] Koroleva A.; Deiwick A.; El-Tamer A.; Koch L.; Shi Y.; Estévez-Priego E.; Ludl A. A.; Soriano J.; Guseva D.; Ponimaskin E.; Chichkov B. In Vitro Development of Human IPSC-Derived Functional Neuronal Networks on Laser-Fabricated 3D Scaffolds. ACS Appl. Mater. Interfaces 2021, 13, 7839–7853. 10.1021/acsami.0c16616. 33559469

[ref5] Harberts R.; Fendler J.; Teuber C.; Siegmund J.; Silva M.; Rieck A.; Wolpert N.; Zierold M.; Blick R. H. Toward Brain-on-a-Chip: Human Induced Pluripotent Stem Cell-Derived Guided Neuronal Networks in Tailor-Made 3d Nanoprinted Microscaffolds. ACS Nano 2020, 14, 13091–13102. 10.1021/acsnano.0c04640. 33058673

[ref6] Aebersold M. J.; Dermutz H.; Forró C.; Weydert S.; Thompson-Steckel G.; Vörös J.; Demkó L. “Brains on a Chip”: Towards Engineered Neural Networks. TrAC, Trends Anal. Chem. 2016, 78, 60–69. 10.1016/j.trac.2016.01.025.

[ref7] Qiu S.; Ji J.; Sun W.; Pei J.; He J.; Li Y.; Li J. J.; Wang G. Recent Advances in Surface Manipulation Using Micro-Contact Printing for Biomedical Applications. Smart Mater. Med. 2021, 2, 65–73. 10.1016/j.smaim.2020.12.002.

[ref8] Yang W.; qin Y.; Wang Z.; Yu T.; Chen Y.; Ge Z. Recent Advance in Cell Patterning Techniques: Approaches, Applications and Future Prospects. Sens. Actuators, A 2021, 333, 113229 10.1016/j.sna.2021.113229.

[ref9] Delamarche E.; Pereiro I.; Kashyap A.; Kaigala G. V. Biopatterning: The Art of Patterning Biomolecules on Surfaces. Langmuir 2021, 37, 9637–9651. 10.1021/acs.langmuir.1c00867. 34347483

[ref10] Banerjee A.; Maity S.; Mastrangelo C. H. Nanostructures for Biosensing, with a Brief Overview on Cancer Detection, IoT, and the Role of Machine Learning in Smart Biosensors. Sensors 2021, 21, 1253 10.3390/s21041253. 33578726 PMC7916491

[ref11] Breault-Turcot J.; Masson J. F. Nanostructured Substrates for Portable and Miniature SPR Biosensors. Anal. Bioanal. Chem. 2012, 403, 1477–1484. 10.1007/s00216-012-5963-1. 22526642

[ref12] Kim D. M.; Park J. S.; Jung S. W.; Yeom J.; Yoo S. M. =Biosensing Applications Using Nanostructure-Based Localized Surface Plasmon Resonance Sensors. Sensors 2021, 21, 3191 10.3390/s21093191. 34064431 PMC8125509

[ref13] Wang Z.; Zong S.; Wu L.; Zhu D.; Cui Y. SERS-Activated Platforms for Immunoassay: Probes, Encoding Methods, and Applications. Chem. Rev. 2017, 117, 7910–7963. 10.1021/acs.chemrev.7b00027. 28534612

[ref14] Fruncillo S.; Su X.; Liu H.; Wong L. S. Lithographic Processes for the Scalable Fabrication of Micro- And Nanostructures for Biochips and Biosensors. ACS Sens. 2021, 6, 2002 10.1021/acssensors.0c02704. 33829765 PMC8240091

[ref15] Lau U. Y.; Saxer S. S.; Lee J.; Bat E.; Maynard H. D. Direct Write Protein Patterns for Multiplexed Cytokine Detection from Live Cells Using Electron Beam Lithography. ACS Nano 2016, 10, 723–729. 10.1021/acsnano.5b05781. 26679368 PMC4729597

[ref16] Liu G.; Petrosko S. H.; Zheng Z.; Mirkin C. A. Evolution of Dip-Pen Nanolithography (DPN): From Molecular Patterning to Materials Discovery. Chem. Rev. 2020, 120, 6009–6047. 10.1021/acs.chemrev.9b00725. 32319753

[ref17] Lucío M. I.; Montoto A. H.; Fernández E.; Alamri S.; Kunze T.; Bañuls M. J.; Maquieira Á. Label-Free Detection of C-Reactive Protein Using Bioresponsive Hydrogel-Based Surface Relief Diffraction Gratings. Biosens. Bioelectron. 2021, 193, 113561 10.1016/j.bios.2021.113561. 34416432

[ref18] Barbulovic-Nad I.; Lucente M.; Sun Y.; Zhang M.; Wheeler A. R.; Bussmann M. Bio-Microarray Fabrication Techniques - A Review. Crit. Rev. Biotechnol. 2006, 26, 237–259. 10.1080/07388550600978358. 17095434

[ref19] Bhatt M.; Shende P. Surface Patterning Techniques for Proteins on Nano- and Micro-Systems: A Modulated Aspect in Hierarchical Structures. J. Mater. Chem. B 2022, 10, 1176–1195. 10.1039/d1tb02455h. 35119060

[ref20] Sancho-Fornes G.; Avella-Oliver M.; Carrascosa J.; Morais S.; Puchades R.; Maquieira Á. Enhancing the Sensitivity in Optical Biosensing by Striped Arrays and Frequency-Domain Analysis. Sens. Actuators, B 2019, 281, 432–438. 10.1016/j.snb.2018.10.130.

[ref21] Alom Ruiz S.; Chen C. S. Microcontact Printing: A Tool to Pattern. Soft Matter 2007, 3, 168–177. 10.1039/b613349e. 32680260

[ref22] Perl A.; Reinhoudt D. N.; Huskens J. Microcontact Printing: Limitations and Achievements. Adv. Mater. 2009, 21, 2257–2268. 10.1002/adma.200801864.

[ref23] Juste-Dolz A.; Avella-Oliver M.; Puchades R.; Maquieira A. Indirect Microcontact Printing to Create Functional Patterns of Physisorbed Antibodies. Sensors 2018, 18, 3163 10.3390/s18093163. 30235856 PMC6164925

[ref24] Correia M.; Snabe T.; Thiagarajan V.; Petersen S. B.; Campos S. R. R.; Baptista A. M.; Neves-Petersen M. T. Photonic Activation of Plasminogen Induced by Low Dose UVB. PLoS One 2015, 10, e0144794 10.1371/journal.pone.0144794. 25635856 PMC4312030

[ref25] Heinz W. F.; Hoh M.; Hoh J. H. Laser Inactivation Protein Patterning of Cell Culture Microenvironments. Lab Chip 2011, 11, 3336–3346. 10.1039/c1lc20204a. 21858278

[ref26] Li Y.; Hong M. Parallel Laser Micro/Nano-Processing for Functional Device Fabrication. Laser Photon. Rev. 2020, 14, 1900062 10.1002/lpor.201900062.

[ref27] Mulko L.; Soldera M.; Lasagni A. F. Structuring and Functionalization of Non-Metallic Materials Using Direct Laser Interference Patterning: A Review. Nanophotonics 2022, 11, 203–240. 10.1515/nanoph-2021-0591. PMC11501624 39633888

[ref28] He J.; Xu B.; Xu X.; Liao C.; Wang Y. Review of Femtosecond-Laser-Inscribed Fiber Bragg Gratings: Fabrication Technologies and Sensing Applications. Photonic Sens. 2021, 11, 203–226. 10.1007/s13320-021-0629-2.

[ref29] Juste-Dolz A.; Delgado-Pinar M.; Avella-Oliver M.; Fernández E.; Pastor D.; Andrés M. V.; Maquieira Á. BIO Bragg Gratings on Microfibers for Label-Free Biosensing. Biosens. Bioelectron. 2021, 176, 112916 10.1016/j.bios.2020.112916. 33401145

[ref30] Gatterdam V.; Frutiger A.; Stengele K.-P.; Heindl D.; Lübbers T.; Vörös J.; Fattinger C. Focal Molography Is a New Method for the in Situ Analysis of Molecular Interactions in Biological Samples. Nat. Nanotechnol. 2017, 12, 1089–1095. 10.1038/nnano.2017.168. 28945239

[ref31] Incaviglia I.; Frutiger A.; Blickenstorfer Y.; Treindl F.; Ammirati G.; Lüchtefeld I.; Dreier B.; Plückthun A.; Vörös J.; Reichmuth A. M. An Approach for the Real-Time Quantification of Cytosolic Protein–Protein Interactions in Living Cells. ACS Sens. 2021, 6, 1572–1582. 10.1021/acssensors.0c02480. 33759497

[ref32] Goh J. B.; Loo R. W.; Goh M. C. Label-Free Monitoring of Multiple Biomolecular Binding Interactions in Real-Time with Diffraction-Based Sensing. Sens. Actuators, B 2005, 106, 243–248. 10.1016/j.snb.2004.08.003.

[ref33] Zhou Z.; Shi Z.; Cai X.; Zhang S.; Corder S. G.; Li X.; Zhang Y.; Zhang G.; Chen L.; Liu M.; Kaplan D. L.; Omenetto F. G.; Mao Y.; Tao Z.; Tao T. H. The Use of Functionalized Silk Fibroin Films as a Platform for Optical Diffraction-Based Sensing Applications. Adv. Mater. 2017, 29, 1605471 10.1002/adma.201605471. 28195379

[ref34] Avella-Oliver M.; Ferrando V.; Monsoriu J. A.; Puchades R.; Maquieira A. A Label-Free Diffraction-Based Sensing Displacement Immunosensor to Quantify Low Molecular Weight Organic Compounds. Anal. Chim. Acta 2018, 1033, 173–179. 10.1016/j.aca.2018.05.060. 30172323

[ref35] Frutiger A.; Tanno A.; Hwu S.; Tiefenauer R. F.; Vörös J.; Nakatsuka N. Nonspecific Binding - Fundamental Concepts and Consequences for Biosensing Applications. Chem. Rev. 2021, 121, 8095–8160. 10.1021/acs.chemrev.1c00044. 34105942

[ref36] Xiong Z.; Peng G. D.; Wu B.; Chu P. L. Effects of the Zeroth-Order Diffraction of a Phase Mask on Bragg Gratings. J. Light. Technol. 1999, 17, 2361–2365. 10.1109/50.803031.

[ref37] Parracino A.; Gajula G. P.; di Gennaro A. K.; Correia M.; Neves-Petersen M. T.; Rafaelsen J.; Petersen S. B. Photonic Immobilization of Bsa for Nanobiomedical Applications: Creation of High Density Microarrays and Superparamagnetic Bioconjugates. Biotechnol. Bioeng. 2011, 108, 999–1010. 10.1002/bit.23015. 21125586

[ref38] Bujacz A. Structures of Bovine, Equine and Leporine Serum Albumin. Acta Crystallogr., Sect. D: Biol. Crystallogr. 2012, 68, 1278–1289. 10.1107/s0907444912027047. 22993082

[ref39] Goodman J. W. Introduction to Fourier Optics, 4th ed.; WH Freeman, 2017.

[ref40] Visentin J.; Couzi L.; Dromer C.; Neau-Cransac M.; Guidicelli G.; Veniard V.; Coniat K. N.; Merville P.; Di Primo C.; Taupin J. L. Overcoming Non-Specific Binding to Measure the Active Concentration and Kinetics of Serum Anti-HLA Antibodies by Surface Plasmon Resonance. Biosens. Bioelectron. 2018, 117, 191–200. 10.1016/j.bios.2018.06.013. 29902635

[ref41] Sancho-Fornes G.; Avella-Oliver M.; Carrascosa J.; Puchades R.; Maquieira Á. Interferometric Multilayered Nanomaterials for Imaging Unlabeled Biorecognition Events. Sens. Actuators, B 2021, 331, 129289 10.1016/j.snb.2020.129289.

