# Supplementary material for: Denaturing for Nanoarchitectonics: Local and Periodic UV-Laser Photodeactivation of Protein Biolayers to Create Functional Patterns for Biosensing
Source: ACS Appl Mater Interfaces. 2022 Sep 1;14(36):41640–8. doi: 10.1021/acsami.2c12808 (PMC9940103; doi:10.1021/acsami.2c12808)
Supplement: Supplementary file 1 — am2c12808_si_001.pdf [file am2c12808_si_001.pdf]

Supporting Information

Denaturing for Nanoarchitectonics: Local and Periodic UV-laser Photodeactivation of Protein Biolayers to Create Functional Patterns for Biosensing

Augusto Juste-Dolz,[1] Martina Delgado-Pinar,[2,*] Miquel Avella-Oliver,[1,3,*] Estrella Fernández,[1] Jose Luís Cruz,[2] Miguel V. Andrés,[2] Ángel Maquieira[1,3,*]

[1] Instituto Interuniversitario de Investigación de Reconocimiento Molecular y Desarrollo Tecnológico (IDM), Universitat Politècnica de València, Universitat de València, 46022 Valencia, Spain.

[2] Department of Applied Physics and Electromagnetism-ICMUV, Universitat de València, 46100 Burjassot, Spain.

[3] Departamento de Química, Universitat Politècnica de València, 46022 Valencia, Spain.

** Corresponding email: amaquieira@qim.upv.es (Á. Maquieira), miavol@upv.es (M. Avella-Oliver), Martina.Delgado@uv.es (M. Delgado-Pinar).*

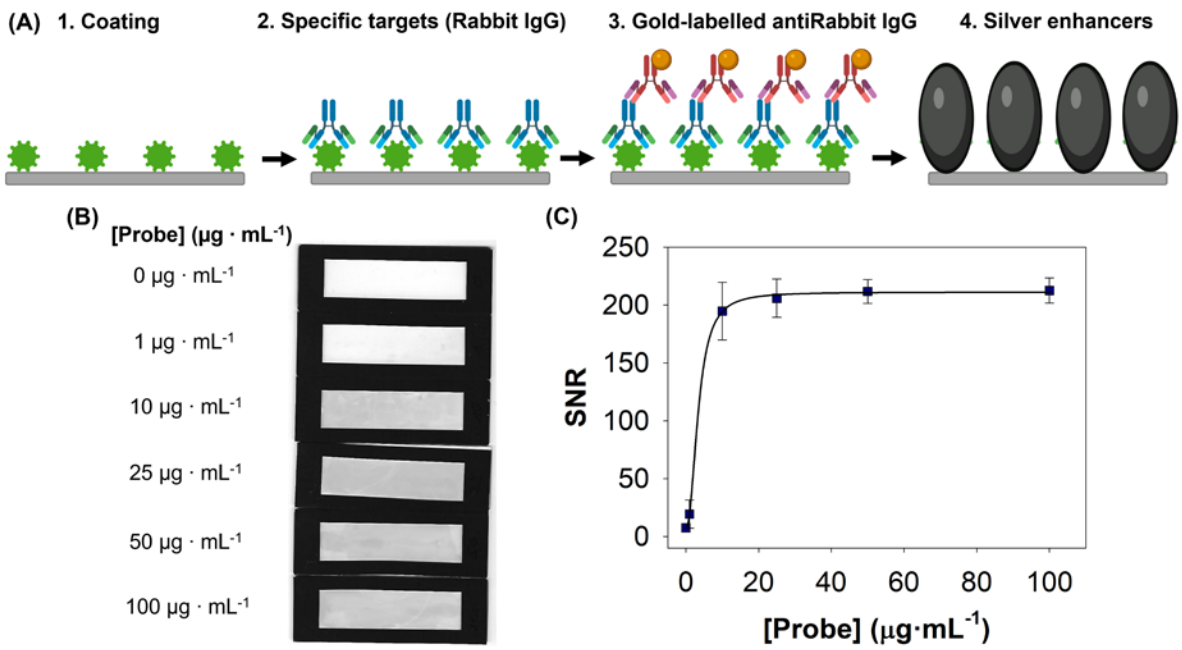

Figure S1. Optimization of the concentration of bioreceptors for surface coating. **(A)** Scheme of the biorecognition assays performed. First, glass slides were coated with different concentrations of BSA (0-100 µg·mL^{-1}) and then incubated with a fixed concentration (10 µg·mL^{-1}) of specific IgGs produced in rabbit. Next, gold-labelled antiRabbit IgGs were incubated to promote the precipitation of metallic silver from a silver solution. **(B)** Coating concentrations and scanned images of the silver-coated slides. **(C)** Signal-to-noise ratios calculated after quantifying the mean grayscale intensity from the scanned images.

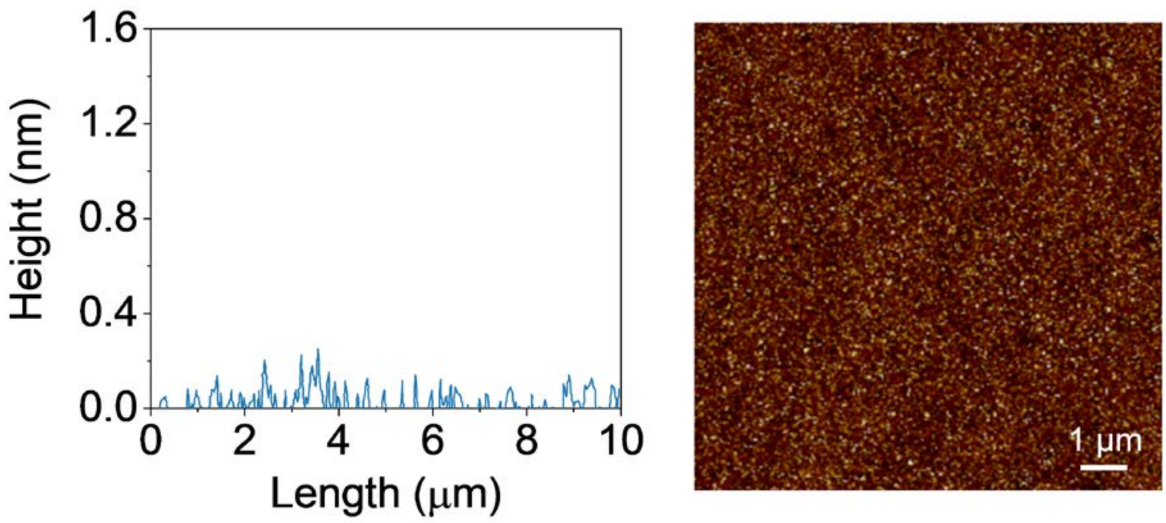

Figure S2. AFM image and height profile of a protein-coated slide after photopatterning with a medium fluence (55 mW, 0.022 cm·s^{-1}).

(A)

10	20	30	40	50
MKWVTFISLL	LLFSSAYSRG	VFRRDTHKSE	IAHRFKDLGE	EHFKGLVLIA
60	70	80	90	100
FSQYLQQCPF	DEHVKLVNEL	TEFAKTCVAD	ESHAGCEKSL	HTLFGDELCK
110	120	130	140	150
VASLRETYGD	MADCCEKQEP	ERNECFLSHK	DDSPDLPKLK	PDPNTLCDEF
160	170	180	190	200
KADEKKFWGK	YLYEIARRHP	YFYAPELLYY	ANKYNGVFQE	CCQAEDKGAC
210	220	230	240	250
LLPKIETMRE	KVLASSARQR	LRCASIQKFG	ERALKAWSVA	RLSQKFPKAE
260	270	280	290	300
FVEVTKLVTD	LTKVHKECCH	GDLLECADDR	ADLAKYICDN	QDTISSKLKE
310	320	330	340	350
CCDKPLLEKS	HCIAEVEKDA	IPENLPPLTA	DFAEDKDVCK	NYQEAKDAFL
360	370	380	390	400
GSFLYEYSRR	HPEYAVSVLL	RLAKEYEATL	EECCAKDDPH	ACYSTVFDKL
410	420	430	440	450
KHLVDEPQNL	IKQNCDQFEK	LGEYGFQNAL	IVRYTRKVPQ	VSTPTLVEVS
460	470	480	490	500
RSLGKVGTRC	CTKPESERMP	CTEDYLSLIL	NRLCVLHEKT	PVSEKVTKCC
510	520	530	540	550
TESLVNRRPC	FSALTPDETY	VPKAFDEKLF	TFHADICTLP	DTEKQIKKQT
560	570	580	590	600
ALVELLKHKP	KATEEQLKTV	MENFVAFVDK	CCAADDKEAC	FAVEGPKLVV
607				
STQTALA				

(B)

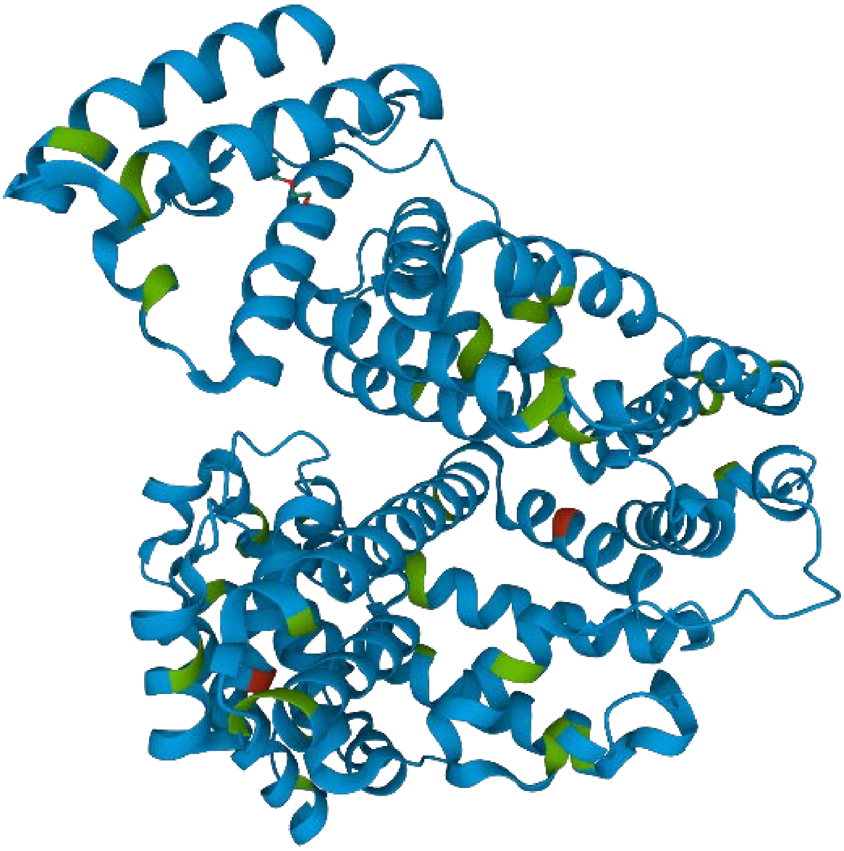

Figure S3. (A) Amino acid sequence of the BSA.[1,2] **(B)** Three-dimensional conformation of the BSA (protein data bank entry 4F5S). In both figures cysteines are represented in green and tryptophans in red color.

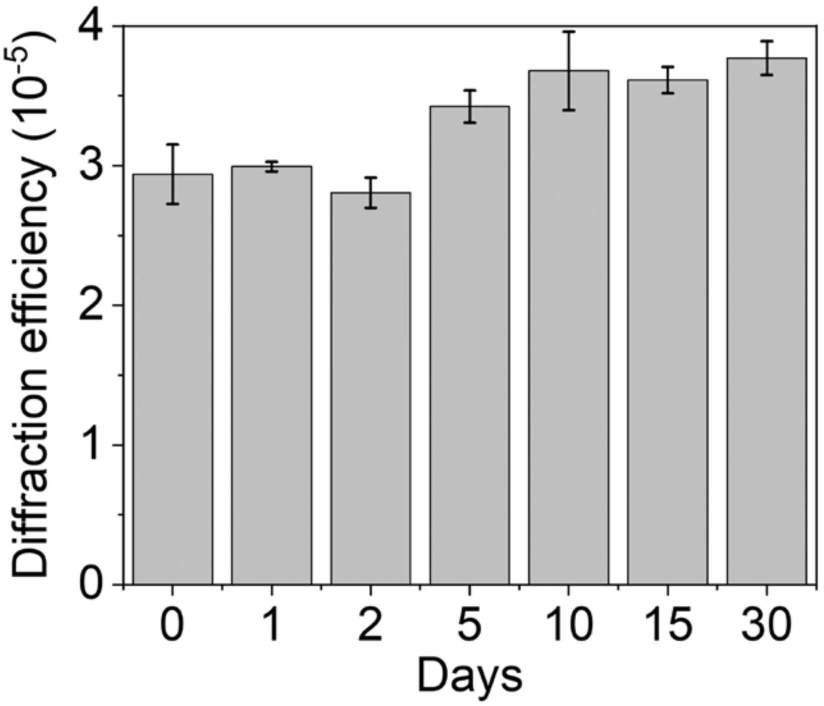

Figure S4. Stability over time. In this experiment, all the BSA patterns were fabricated at the same time (day 0). Then, the diffraction efficiency after incubating specific IgG (antiBSA, 10 µg·mL^{-1} in buffer) was measured after different days.

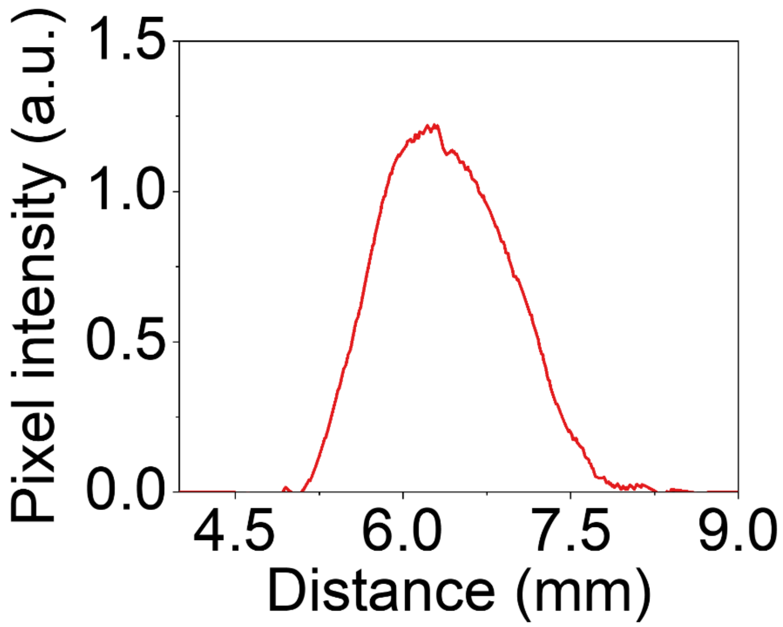

Figure S5. Zoomed view of the cross-section profile of the first-order diffracted spots for a photopatterned BSA biolayer after the incubation of 0 µg mL^{-1} of antiBSA. Note the difference in the vertical scale versus Figure 3C in the main manuscript.

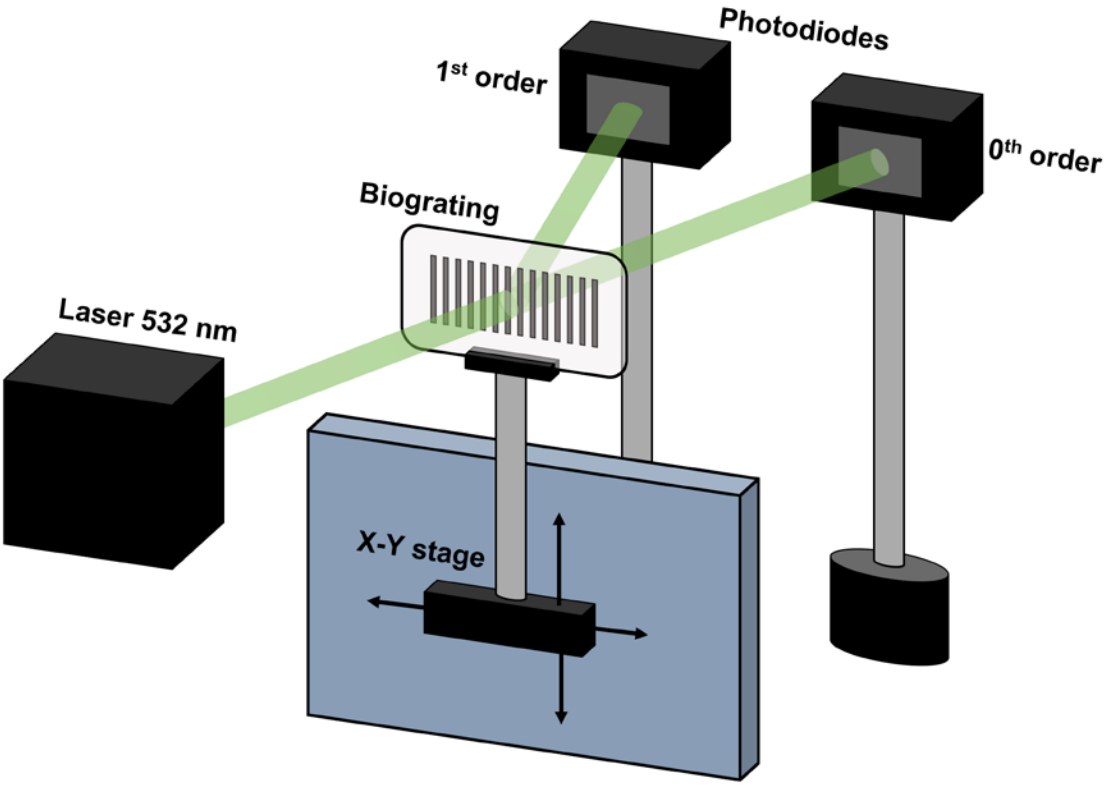

Figure S6. Scheme of the optical setup employed to map the diffraction efficiency of the nanostructures along the patterned area. Glass slides containing the protein patterns were placed in a custom X-Y stage with minimum displacement of 0.5 mm and then irradiated with a 532 nm laser source. The intensity of the zeroth and first diffracted orders was measured employing two photodiodes.

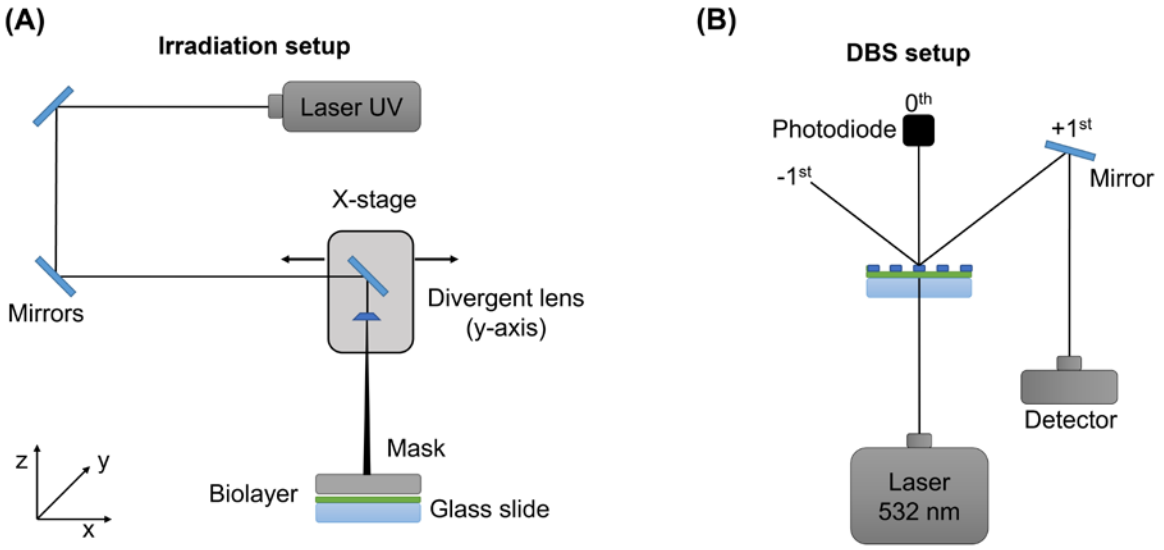

Figure S7. Schemes of **(A)** the irradiation setup for selective protein deactivation and **(B)** the optical setup to quantify the diffraction efficiency of the nanostructures.

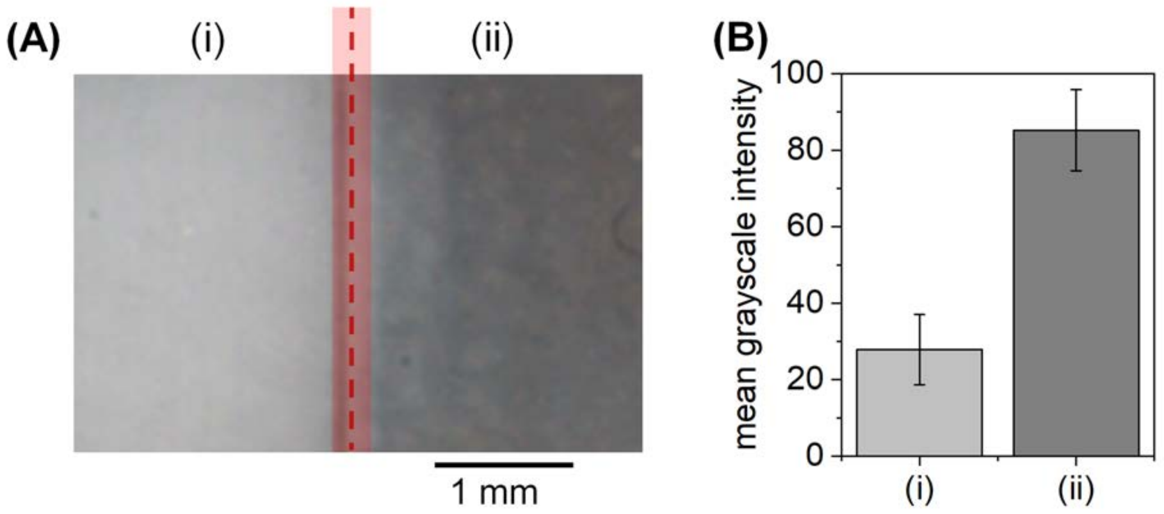

Figure S8. **(A)** Scaned image and **(B)** the resulting grayscale intensity of a BSA biolayer (i) irradiated with a strong fluence (about 66 J·cm^{-2}) and (ii) not irradiated, after perfoming the gold-labelled immunoassay described in the legend of Figure S1. Note that an important contribution of the grayscale intensity measured in the irradiated area may be generated by inspecific precipitation of silver in the signal development stage of this labelled assay.

Table S1. Fabrication conditions and topographic features of the protein patterns measured by AFM.

	**Fluence (J·cm^{-2})**	**Laser power (mW)**	**Motion velocity (cm·s^{-1})**	**Height modulation (nm)**	**Period (nm)**	**Duty cycle (%)**
Low	0.1	55	0.44	0.49 ± 0.10	711 ± 3	60 ± 3
Medium	2.5	55	0.022	1.25 ± 0.13	709 ± 2	49 ± 3
High	9.9	55	0.011	0.31 ± 0.12	710 ± 2	38 ± 6

Table S2. Comparative table of recent diffractive and non-diffractive label-free biosensing approaches in the state-of-art.

**Technique**	**Target**	**Limit of detection**	**Matrix**	**Reference**
SPR	ssDNA	0.1 nM	Buffer	3
SPR	kanamycin	285 nM	Buffer	4
SPR	HSA	100 ng·mL^{-1}	Buffer	5
Focal molography	IgG	1.3 nM	Human plasma	6
Diffractive hydrogels	CRP	300 ng·mL^{-1}	Human serum	7
Diffractive reflectance	streptavidin	25 nM	Buffer	8
Bio Bragg Gratings	IgG	100 ng·mL^{-1}	Buffer	9
Diffraction-based sensing	IgG	53 ng·mL^{-1}/ 0.4 nM	Buffer	This work
Diffraction-based sensing	IgG	36 ng·mL^{-1}/ 0.3 nM	Human serum	This work

References

(1) Parracino, A.; Gajula, G. P.; di Gennaro, A. K.; Correia, M.; Neves-Petersen, M. T.; Rafaelsen, J.; Petersen, S. B. Photonic Immobilization of Bsa for Nanobiomedical Applications: Creation of High Density Microarrays and Superparamagnetic Bioconjugates. *Biotechnol. Bioeng.* **2011**, *108* (5), 999–1010. https://doi.org/10.1002/bit.23015.

(2) Bujacz, A. Structures of Bovine, Equine and Leporine Serum Albumin. *Acta Crystallogr. Sect. D Biol. Crystallogr.* **2012**, *68* (10), 1278–1289. https://doi.org/10.1107/S0907444912027047.

(3) An, N.; Li, K.; Zhang, Y.; Wen, T.; Liu, W.; Liu, G.; Li, L.; Jin, W. A Multiplex and Regenerable Surface Plasmon Resonance (MR-SPR) Biosensor for DNA Detection of Genetically Modified Organisms. *Talanta* **2021**, *231*, 122361. https://doi.org/10.1016/j.talanta.2021.122361.

(4) Écija-Arenas, Á.; Kirchner, E.-M.; Hirsch, T.; Fernández-Romero, J. M. Development of an Aptamer-Based SPR-Biosensor for the Determination of Kanamycin Residues in Foods. *Anal. Chim. Acta* **2021**, *1169*, 338631. https://doi.org/10.1016/j.aca.2021.338631.

(5) Makhneva, E.; Farka, Z.; Pastucha, M.; Obrusník, A.; Horáčková, V.; Skládal, P.; Zajíčková, L. Maleic Anhydride and Acetylene Plasma Copolymer Surfaces for SPR Immunosensing. *Anal. Bioanal. Chem.* **2019**, *411* (29), 7689–7697. https://doi.org/10.1007/s00216-019-01979-9.

(6) Gatterdam, V.; Frutiger, A.; Stengele, K.-P.; Heindl, D.; Lübbers, T.; Vörös, J.; Fattinger, C. Focal Molography Is a New Method for the in Situ Analysis of Molecular Interactions in Biological Samples. *Nat. Nanotechnol.* **2017**, *12* (11), 1089–1095. https://doi.org/10.1038/nnano.2017.168.

(7) Lucío, M. I.; Montoto, A. H.; Fernández, E.; Alamri, S.; Kunze, T.; Bañuls, M. J.; Maquieira, Á. Label-Free Detection of C-Reactive Protein Using Bioresponsive Hydrogel-Based Surface Relief Diffraction Gratings. *Biosens. Bioelectron.* **2021**, *193* (August). https://doi.org/10.1016/j.bios.2021.113561.

(8) Chen, W. T.; Li, S. S.; Chu, J. P.; Feng, K. C.; Chen, J. K. Fabrication of Ordered Metallic Glass Nanotube Arrays for Label-Free Biosensing with Diffractive Reflectance. *Biosens. Bioelectron.* **2018**, *102* (September 2017), 129–135. https://doi.org/10.1016/j.bios.2017.10.023.

(9) Juste-Dolz, A.; Delgado-Pinar, M.; Avella-Oliver, M.; Fernández, E.; Pastor, D.; Andrés, M. V.; Maquieira, Á. BIO Bragg Gratings on Microfibers for Label-Free Biosensing. *Biosens. Bioelectron.* **2021**, *176* (December 2020). https://doi.org/10.1016/j.bios.2020.112916.
